# The Nitric Oxide Donor [Zn(PipNONO)Cl] Exhibits Antitumor Activity through Inhibition of Epithelial and Endothelial Mesenchymal Transitions

**DOI:** 10.3390/cancers14174240

**Published:** 2022-08-31

**Authors:** Valerio Ciccone, Arianna Filippelli, Chiara Bacchella, Enrico Monzani, Lucia Morbidelli

**Affiliations:** 1 Department of Life Sciences, University of Siena, Via A. Moro 2, 53100 Siena, Italy; 2 Department of Experimental Medicine, University of Campania “Luigi Vanvitelli”, Via Santa Maria di Costantinopoli 16, 80138 Naples, Italy; 3 Department of Chemistry, University of Pavia, Via Taramelli 12, 27100 Pavia, Italy; 4 Noxamet Limited Company, Via Besana 2, 20122 Milan, Italy

**Keywords:** nitric oxide, metal nonoate, tumor cell, endothelial cell, epithelial–mesenchymal transition, endothelial–mesenchymal transition, transforming growth factor beta

## Abstract

**Simple Summary:**

Nitric oxide (NO) plays a critical pathophysiological role in cancer by modulating several processes, such as angiogenesis, tumor growth, and metastatic potential. The aim of this study was to characterize the antitumor effects of a novel NO donor, [Zn(PipNONO)Cl], on the processes of epithelial– and endothelial–mesenchymal transitions (EMT and EndMT), known to actively participate in cancer progression. Two tumor cells lines were used in this study: human lung cancer cells (A549) and melanoma cells (A375), alone and co-cultured with human endothelial cells. Our results demonstrate that both tumor and endothelial cells were targets of NO action, which impaired EMT and EndMT functional and molecular features. Further studies are needed to finalize the therapeutic use of the novel NO donor.

**Abstract:**

Exogenous nitric oxide appears a promising therapeutic approach to control cancer progression. Previously, a nickel-based nonoate, [Ni(SalPipNONO)], inhibited lung cancer cells, along with impairment of angiogenesis. The Zn(II) containing derivatives [Zn(PipNONO)Cl] exhibited a protective effect on vascular endothelium. Here, we have evaluated the antitumor properties of [Zn(PipNONO)Cl] in human lung cancer (A549) and melanoma (A375) cells. Metastasis initiates with the epithelial–mesenchymal transition (EMT) process, consisting of the acquisition of invasive and migratory properties by tumor cells. At not cytotoxic levels, the nonoate significantly impaired A549 and A375 EMT induced by transforming growth factor-β1 (TGF-β1). Reduction of the mesenchymal marker vimentin, upregulated by TGF-β1, and restoration of the epithelial marker E-cadherin, reduced by TGF-β1, were detected in both tumor cell lines in the presence of Zn-nonoate. Further, the endothelial–mesenchymal transition achieved in a tumor-endothelial cell co-culture was assessed. Endothelial cells co-cultured with A549 or A375 acquired a mesenchymal phenotype with increased vimentin, alpha smooth muscle actin and Smad2/3, and reduced VE-cadherin. The presence of [Zn(PipNONO)Cl] maintained a typical endothelial phenotype. In conclusion, [Zn(PipNONO)Cl] appears a promising therapeutic tool to control tumor growth and metastasis, by acting on both tumor and endothelial cells, reprogramming the cells toward their physiologic phenotypes.

## 1. Introduction

The gaseous transmitter nitric oxide (NO) is widely recognized to play key roles in various physiological and pathological processes. The effect of NO on tumor growth clearly demonstrates NO’s innate bimodal activity, depending on its concentration. Low NO levels promote tumor cell proliferation and migration and angiogenesis; and high concentrations (≥0.5 mM) are recognized to exert anti-tumor effects by promoting DNA damage and gene mutations, protein dysfunction and cell death through apoptosis [1,2,3]. Exogenously given NO or NO promoting approaches have been demonstrated to inhibit tumor growth, impair tumor angiogenesis and control metastatic potential [4,5].

Various pharmaceutical approaches have been developed and tested for antitumor activities [6,7], and NO alone or complexed with reactive oxygen species induces killing or sensitizes a range of different types of cancer [8,9] to chemo-, radio- and immunotherapies [10,11,12,13].

There is increasing interest in developing NO-releasing compounds that are able to generate NO in particular tissues, avoiding systemic toxic effects. Among the many NO donors, the nonoates (also known as diazeniumdiolates) consist of a diolate moiety attached to a primary or secondary amine or polyamine. They are stable in solid form, but hydrolyze spontaneously in solution at physiological temperature and pH to generate two equivalents of NO per molecule [14]. The efficacy and safety of a series of metal nonoates have already been assessed in different experimental models of cardiovascular diseases [15,16]. In particular, the Zn(II) containing derivative [Zn(PipNONO)Cl] exhibited interesting kinetics of NO release (t1/2 385 s). This compound elicited a protective effect on normal endothelium and smooth muscle cells, promoting vasorelaxation and increased bioavailability of H_2_S, which concurs with the protective effects [17]. Among the metal nonoates, the nickel-based compound [Ni(SalPipNONO)] exhibited antitumor activity in lung cancer cells, through the impairment of tumor angiogenesis [18]. Based on the limitations due to the presence of nickel, in this paper we focus our attention on a zinc-containing nonoate.

Among the multiple cellular and biochemical mechanisms at the basis of cancer progression and metastasis, the phenomena of epithelial–mesenchymal transition (EMT) and endothelial–mesenchymal transition (EndMT) are focuses for the development of novel anticancer therapeutics [19,20].

EMT characterizes metastasis, where normal cell–cell adhesion between epithelial cells is lost in tandem with the gain of mesenchymal markers, thereby promoting cell migration and invasion [21,22,23]. An inflammatory tumor microenvironment facilitates EMT, i.e., through the upregulation of transforming growth factor-beta 1 (TGF-β1) and its receptor signaling [24,25]. EMT is accompanied by epigenetic reprogramming, which leads to the loss of typical epithelial markers such as E-cadherin, and the acquisition of mesenchymal markers such as vimentin and alpha smooth muscle actin (αSMA) [22,23].

While low/moderate NO levels promote EMT and metastasis [5], high levels of NO, delivered through NO donors such as diethylenetriamine nonoate (DETA/NO), were demonstrated to inhibit the EMT process in metastatic cancer cell lines by downregulating the NF-κB signaling [26,27]. Consistently, other reports showed that NO facilitated the induction of mesenchymal epithelial transition and cell aggregation in malignant cells [28].

Sustained inflammation acts also in the endothelial component [29]. Endothelial mesenchymal transition (EndMT) is defined as the loss of endothelial-specific markers and the gain of mesenchymal features that accompany the morphogenesis of specific tissues, especially those of the heart [30,31]. Long-term exposure to inflammatory cytokines can induce EndMT by changing the morphology of endothelial cells from cuboidal to a spindle and acquiring EndMT-linked markers [32]. Besides the shifting in the expression of endothelial toward mesenchymal markers, inflammatory cytokines can ignite the TGF-β signaling and other pathways [29]. At the molecular level, EndMT is accompanied by decreased expression of markers typical of endothelial cells, such as CD31 (or platelet endothelial cell adhesion molecule-1), CD34, vascular endothelial cadherin (VE-cadherin) and von Willebrand factor; the expression of mesenchymal cell markers (i.e., α-SMA and vimentin) rises with increased cell migration [33,34].

EndMT occurs in various pathologic conditions, including cancer progression [35]. The conversion of endothelial cells into mesenchymal-lineage cell types, especially myofibroblasts, occurs in an aberrant manner, contributing to disease progression [20,31,35,36,37]. Indeed, tumor endothelial cells appear constitutively prone to mesenchymal transition. The EndMT phenomenon is gradual and multistep, and till a certain point, reversible [38].

Among the tumors which afflict humans, effective therapeutic interventions for lung carcinoma and melanoma remain unresolved medical needs. In particular, non-small cell lung carcinoma (NSCL) and malignant melanoma share poor prognosis, and the therapeutic options are few and characterized by scarce effectiveness and resistance [39,40]. These malignancies show a predisposition to EMT and EndMT [41,42,43,44]. Few are the reports on the development of NO donors in these tumor types [18,45,46,47,48]; thus, the field merits investigation.

Beyond its antiproliferative capacity, we hypothesize that [Zn(PipNONO)Cl] regulates some crucial steps of the metastasis process, as TGF-β1 induced EMT in tumor cells and EndMT in endothelial cells co-cultured with cancer cells.

## 2. Materials and Methods

### 2.1. Cell Cultures

The human cell line A549, representative of non-small cell lung cancer, and A375, representative of metastatic human melanoma cells, were purchased from American Type Culture Collection. Tumor cells were cultured in DMEM 4500 high glucose (Euroclone SpA, Milan, Italy) supplemented with 10% fetal bovine serum (FBS; HyClone; Cytiva, Marlborough, MA, USA) and 2 mM glutamine, 100 units/mL penicillin and 0.1 mg/mL streptomycin (Merck KGaA, Darmstadt, Germany). Tumor cells were cultured in 10 cm diameter Petri dishes up to a confluent state, in a humidified atmosphere with 5% CO_2_. Cells were expanded through splitting 1:4 or 1:5 twice a week and used until passage 30.

Human umbilical vein endothelial cells (HUVECs) were purchased from Promocell (Heidelberg, Germany). Endothelial growth medium (EGM-2) contained the following factors: vascular endothelial growth factor (VEGF), human fibroblast growth factor (hFGF), recombinant human long R3 insulin-like growth factor 1 (R3-IGF-1), human epidermal growth factor (hEGF), ascorbic acid, hydrocortisone, heparin GA-1000 (Lonza, Basel, Switzerland), 2 mM glutamine, 100 units/mL penicillin and 0.1 mg/mL streptomycin (Merck KGaA, Darmstadt, Germany). HUVECs were cultivated on 1% bovine gelatin coated dishes with complete EGM-2 supplemented with 10% FBS and propagated once a week by 1:3 dilution.

### 2.2. Survival Assay

Cell survival was quantified by MTT assay [18]. First, 2 × 10^3^ tumor cells were seeded in 96-multiwell plates in medium with 10% FBS and after adherence were exposed to increasing concentrations of [Zn(PipNONO)Cl] (provided by Noxamet Ltd., Milan, Italy). [Zn(PipNONO)Cl] was dissolved in DMSO at 100 mM and further diluted in medium before the addition to the cells. A concentration of DMSO equal to the highest concentration used in each experiment was used as the control. After 24, 48, 72 and 96 h, medium was removed and cells were incubated for 4 h with fresh medium in the presence of 1.2 mM 3-(4,5-dimethylthiazol-2-yl)-2,5-diphenyltetrazolium bromide (MTT) (Sigma-Aldrich, St. Louis, MO, USA). After solubilization of formazan crystals in DMSO, absorbance was measured with a microplate absorbance reader (Infinite 200 Pro, Tecan Life Sciences, Männedorf, Switzerland) at 540 nm. Data are reported as fold changes of 540 nm relative absorbance/well, with respect to the basal control.

### 2.3. HUVEC Proliferation Assay

HUVECs (1.5 × 10^3^ cells/well in 96 multiplates), after adherence, were treated with [Zn(PipNONO)Cl] (10 µM and 50 µM) for 48 h. Cells were then fixed, stained and randomly counted at 20× original magnification in 5 fields, as previously reported [49].

### 2.4. Co-Culturing of HUVECs with Cancer Cells and EndMT Protein Detection

#### 2.4.1. Co-Culture Setup

Intercellular communication between neoplastic and endothelial cells was reconstructed in vitro using transwell technology (Corning, Lowell, MA, USA). Transwells allow co-culturing between two cell types without direct contact. HUVECs (1.5 × 10^5^ cells) were seeded on the bottoms of 6-well multi-plates pre-coated with gelatin. Tumor cells (8 × 10^4^ cells) were plated on the polyester membrane of the Transwell [50]. After 24 h alone, tumor cells were transferred into the same 6-well multiplates containing HUVECs on the bottom. Co-culture continued for 4 days in the presence of EBM medium (without growth factors) with 2% FBS. Where indicated, co-cultures were treated with 50 µM of [Zn(PipNONO)Cl], readded every 48 h.

#### 2.4.2. Western Blot

Proteins from HUVEC monolayers were isolated, and Western blotting was performed as previously described [50]. Separated proteins were then blotted onto nitrocellulose membranes and incubated overnight with primary antibodies. Anti-vimentin (rabbit, 1:1000, catalog number 5741S) and anti VE-cadherin (rabbit, 1:1000, catalog number 2500S) were from Cell Signaling Technology, Inc. (Danvers, MA, USA). Anti-αSMA (mouse, 1:1000, catalog number A5228) was from Merck KGaA (Darmstadt, Germany). All signals were then detected by an enhanced chemiluminescence system (Biorad, Hercules, CA, USA). Results were normalized to those obtained by using an antibody against β-actin (mouse, 1:10,000, catalog number MABT825 from Merck KGaA, Darmstadt, Germany) and quantified through Fiji ImageJ software (64-bit Java 1.8.0_172, U.S. National Institutes of Health, Bethesda, MD, USA). Data were analyzed as ratio of the arbitrary densitometry unit (A.D.U.) of the target protein to that of the reference protein, and in most of the graphs, are compared to the basal control condition.

#### 2.4.3. Immunofluorescence Analysis

The TGF-β1 signaling molecule Smad2/3 was visualized in HUVECs by immunofluorescence analysis. A total of 3 × 10^4^ HUVECs were seeded on 1 cm circular glass coverslips added in the bottom of a 24 well multiplate. Tumor cells were seeded at a density of 2 × 10^4^ on the top of 0.4 μm pore polycarbonate membranes of the transwells. After 24 h of incubation, transwells were put in the same 24 multiplates and co-cultured (in medium with 2% FBS) for 4 days. Where indicated, co-cultures were treated with 50 µM [Zn(PipNONO)Cl], readded every 48 h. Immunofluorescence analysis was performed as previously reported [51] using anti Smad2/3 antibody (rabbit, 1:500, catalog number 8685S) from Cell Signaling Technology, Inc. (Danvers, MA, USA), and DAPI as nuclear counterstaining dye. Images were taken using a confocal microscope (Zeiss LSM700; Zeiss GmbH, Oberkochen, Germany).

### 2.5. Tumor Cell Migration

A scratch healing assay was performed to determine cell migration, as previously described [50]. Tumor cells were seeded in a 24-well plate at a density of 1.4 × 10^5^ cells/well, and after 24 h of growth, they reached confluence as a monolayer. Then, a scratch was made in the center of the well with a micropipette tip. After gentle rinsing, cells were treated with [Zn(PipNONO)Cl] at increasing concentrations (10, 50 and 100 µM) in medium with 2% FBS for 18 h to allow migration. Images of the scratches were captured with a microscope (Nikon Eclipse E400, Tallahassee, FL, USA).

### 2.6. Tumor Cell Invasion Assay

The Neuro Probe 48-well micro-chemotaxis chamber (Nuclepore Corp., Pleasanton, CA, USA) was used to evaluate the effect of [Zn(PipNONO)Cl] on tumor cell invasiveness. The Boyden chamber setup allows one to measure the passage of tumor cells across porous filters from an upper compartment to a lower compartment, according to a concentration gradient of a stimulus [52]. Polyvinylpyrrolidone-free polycarbonate filters, 8 µm pore size (Neuro-Probe, Inc, Gaithersburg, MD, USA), were coated with 1% gelatin (gelatin type 1 from bovine skin, Sigma Aldrich, St. Louis, MO, USA) to mimic the extracellular matrix. Two chemotactic solutions, i.e., medium with 1% and 5% FBS, were placed in the lower wells. Then, 50 µL of cell suspension (2.5 × 10^4^ cells/mL) was added to each upper compartment. Before invasion challenging, tumor cell suspensions were incubated with 100 µM [Zn(PipNONO)Cl] or vehicle at 37 °C for 30 min. Once assembled, the apparatus was incubated at 37 °C. After 18 h, the filter was removed and fixed overnight in methanol. Cells were stained with Diff-Quik (BiomapSnc, Agrate B.za, MI, Italy), and non-migrated cells sedimented on the upper surface of the filter were removed with a cotton swab. The filter was then cut and mounted between glass coverslips. Migrated cells across and under the filter were counted through the use of a light microscope (Nikon Eclipse E400) at 20× magnification: 5 random fields/well. Each experimental condition was performed in triplicate, and invasiveness is reported as the mean value of migrated cells (±SD).

### 2.7. EMT Protein Expression in Tumor Cells

Protein expression was evaluated by Western blot [50]. Tumor cells were seeded at the density of 8 × 10^4^ cells/6 cm on a Petri dish and allowed to adhere for 24 h. Cells were stimulated with TGF-β1 (10 ng/mL) (Merck KGaA, Darmstadt, Germany) in medium with 0.5% for 96 h, while adding the stimulus again every 24 h. Where added, [Zn(PipNONO)Cl] was refreshed at 48 h. At the end of the stimulation, cells were rinsed twice with ice-cold PBS, and lysed with 60 µL of CelLytic (Sigma-Aldrich, St. Louis, MO, USA), along with sodium orthovanadate (Na_3_VO_4_·2H_2_O) (1 mM) and a cocktail of protease inhibitors (Sigma Aldrich, St. Louis, MO, USA). Following extraction and quantification of proteins, electrophoresis was carried out. Fifty micrograms of protein/sample was run in 4–12% Bis-Tris Gels (Life Technologies, Carlsbad, CA, USA). Separated proteins were then blotted onto nitrocellulose membranes, which were incubated overnight with the following primary antibodies: anti-E-Cadherin (rabbit, 1:1000, catalog number 3195S), anti-vimentin (rabbit, 1:1000, catalog number 5741S), anti-TGF-β1 (rabbit, 1:1000, catalog number 3711S) and anti Smad2/3 (rabbit, 1:500, catalog number 8685S), all from Cell Signaling Technology, Inc. (Danvers, MA, USA). The signals were then detected by enhanced chemiluminescence system (Biorad, Hercules, CA, USA). Results were normalized to those obtained by using an antibody against β-actin (mouse, 1:10,000, catalog number MABT825 from Merck KGaA, Darmstadt, Germany) and quantified through Fiji ImageJ software. Data were analyzed as ratio of the arbitrary densitometry unit (A.D.U.) of the target protein with respect to the reference protein, and in most of the graphs, are reported in comparison to the basal control condition.

### 2.8. RNA Isolation and Reverse Transcription-Quantitative Polymerase Chain Reaction (RT-q PCR)

After cell stimulation, total RNA was extracted using an RNeasy Plus kit (catalog number 74134, Qiagen GmbH, Germantown, MD, USA), following the manufacturer’s instructions. The quality and quantity of the purified RNA were determined by measuring the absorbance at 260/280 nm (A260/A280) using Infinite F200 Pro (Tecan Life Sciences, Männedorf, Switzerland). A total of 1 µg of RNA was reverse-transcribed using QuantiTect Reverse Transcription kit (catalog number 205313, Qiagen GmbH, Germantown, MD, USA) at 42 °C for 30 min. The reaction was stopped by incubating the tubes at 95 °C for 3 min. RT-qPCR was carried out by using QuantiNova SYBR Green PCR kit (catalog number 208056, Qiagen GmbH) in a RotorGene qPCR instrument (Qiagen GmbH, Germantown, MD, USA). The following conditions were used: 95 °C for 5 min; followed by 45 cycles of 95 °C for 20 s and 60 °C for 20 s; and then 72 °C for 20 s. The change in gene expression was quantified by the comparative Ct (cycle threshold) method (ΔCt) normalized to the expression of 60S ribosomal protein L19 (RPL19). RT-qPCR data were reported as fold increases in Ct values with respect to untreated cells. The primer sequences were: RPL19 forward 5′-GAT GCC GGA AAA ACA CCT TG-3′. RPL19 reverse: 5′-TGG CTG TAC CCT TCC GCT T-3′. TGF-β1 forward: 5′-AGCAGCACGTGGAGCTGT-3′. TGF-β1 reverse: 5′-CAGCCGGTTGCTGAGGTA-3′. All primers were from Merck KGaA.

### 2.9. Data Analysis and Statistical Procedures

Results are either representative or the averages of at least 3 independent experiments performed in triplicate. Statistical analysis was performed using ANOVA, followed by the Bonferroni test and the Student’s *t* test when appropriate (GraphPad, San Diego, CA, USA). A *p* value < 0.05 was considered statistically significant.

## 3. Results

### 3.1. Effect of [Zn(PipNONO)Cl] on Tumor Cell Viability

**Figure 1:**
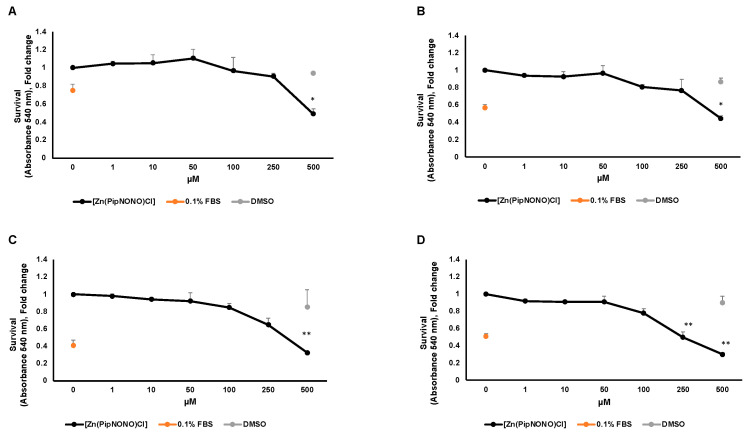

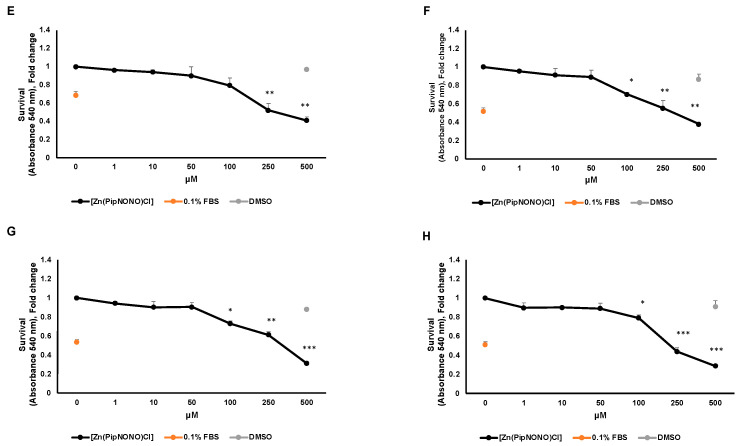
Effect of [Zn(PipNONO)Cl] on tumor cell survival. Dose–response curves of A549 cells treated with [Zn(PipNONO)Cl] after 24 h (**A**), 48 h (**B**), 72 h (**C**) and 96 h (**D**); and those of A375 in the same experimental conditions (**E**–**H**). The graphs show the cell survival response following treatment with [Zn(PipNONO)Cl], used at increasing concentrations (from 1 to 500 μM), in the presence of medium with 10% FBS. The values of the means for each experimental point were normalized on the basal condition of medium with 10% FBS (set arbitrarily equal to 1). Medium with 0.1% FBS was used as a negative control, and DMSO used at a concentration of 0.5% was tested as a vehicle control. * *p* ˂ 0.05, ** *p* ˂ 0.01 and *** *p* < 0.001 vs. control and DMSO.

The antitumor activity of [Zn(PipNONO)Cl] was studied by using the MTT viability assay on A549 and A375 tumor cells. In this experiment, the MTT assay was performed at four times: 24, 48, 72 and 96 h. In order to evaluate the maximal tolerated concentration and identify the dose necessary for a response by the tumor cells, increasing concentrations (1–500 μM) of [Zn(PipNONO)Cl] were tested. Stimulation of tumor cells took place in medium with 10% FBS. As a negative control for cell viability, cells were exposed to medium with 0.1% FBS. DMSO, the solubilization vehicle of [Zn(PipNONO)Cl], was tested at the highest dose corresponding to that of 500 μM of the nonoate. The survival assay documented an inhibitory effect by [Zn(PipNONO)Cl] on tumor growth in a concentration-dependent manner. In particular, doses higher than 100 μM were cytotoxic, and lower concentrations were compatible with cell viability (Figure 1A–D for A549 and Figure 1E–H for A375). Based on these results, concentrations equal or >500 μM were not used in the further functional and molecular experiments.

### 3.2. [Zn(PipNONO)Cl] Inhibited the Migration and Invasion of Tumor Cells

**Figure 2:**
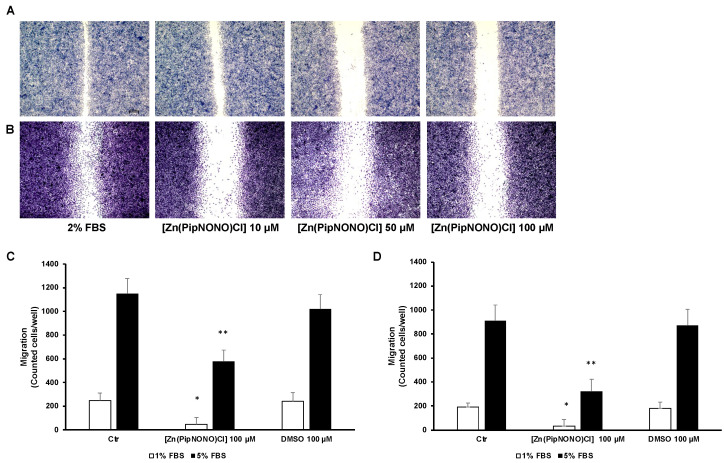
Effect of [Zn(PipNONO)Cl] on the migration and invasiveness of tumor cells. Scratch assay of A549 (**A**) and A375 (**B**) treated with various concentrations (10, 50 and 100 μM) of [Zn(PipNONO)Cl] for 18 h. Representative images taken with 4× magnification. (**C**,**D**) In the invasion assay, A549 (**C**) and A375 (**D**) were exposed to 100 μM [Zn(PipNONO)Cl] or DMSO (0.1%) and then allowed to migrate toward different serum concentrations (1% and 5%, white and black columns, respectively) put in the lower compartments of the Boyden chamber, the porous filter being coated with gelatin. Number in the graphs are counted cells/well. * *p* ˂ 0.05 vs. control and DMSO; ** *p* ˂ 0.01 vs. control and DMSO.

To assess the role of [Zn(PipNONO)Cl] in tumor cell migration and invasive motility, a scratch assay was performed. [Zn(PipNONO)Cl] reduced A549 (Figure 2A) and A375 (Figure 2B) motility compared to untreated cells. The scratch-healing rate of the nonoate treatment group decreased gradually in a dose-dependent manner (Figure 2A,B). Next, we focused on the invasive ability of tumor cells by using the Boyden chamber assay. This invasiveness test allows one to evaluate the ability of tumor cells in suspension to migrate through a porous filter coated with gelatin, thereby mimicking the process of tumor metastasis. This experimental approach was performed considering two different serum concentrations (1 and 5% FBS) used as chemo-attracting stimuli placed in the lower compartments. As expected, the values obtained from our results show how the migration of A549 (Figure 2C) and A375 (Figure 2D) with 1% FBS is lower compared to the values found with 5% FBS placed in the lower compartment (Figure 2C,D). Tumor cells were exposed to [Zn(PipNONO)Cl] at a concentration of 100 μM before being tested for their migratory capacities. In the presence of 1% FBS, the number of migrating tumor cells after exposure to the metal nonoate was significantly reduced (to one fifth of the basal control and of the DMSO control). When cell migration was challenged towards 5% FBS, the incubation of cells with [Zn(PipNONO)Cl] halved the migratory capacity compared to the baseline control and DMSO alone (Figure 2A,B). Overall, the data indicate an inhibitory effect on tumor migration by the zinc-based nonoate.

### 3.3. Effect of [Zn(PipNONO)Cl] on EMT Markers

**Figure 3:**
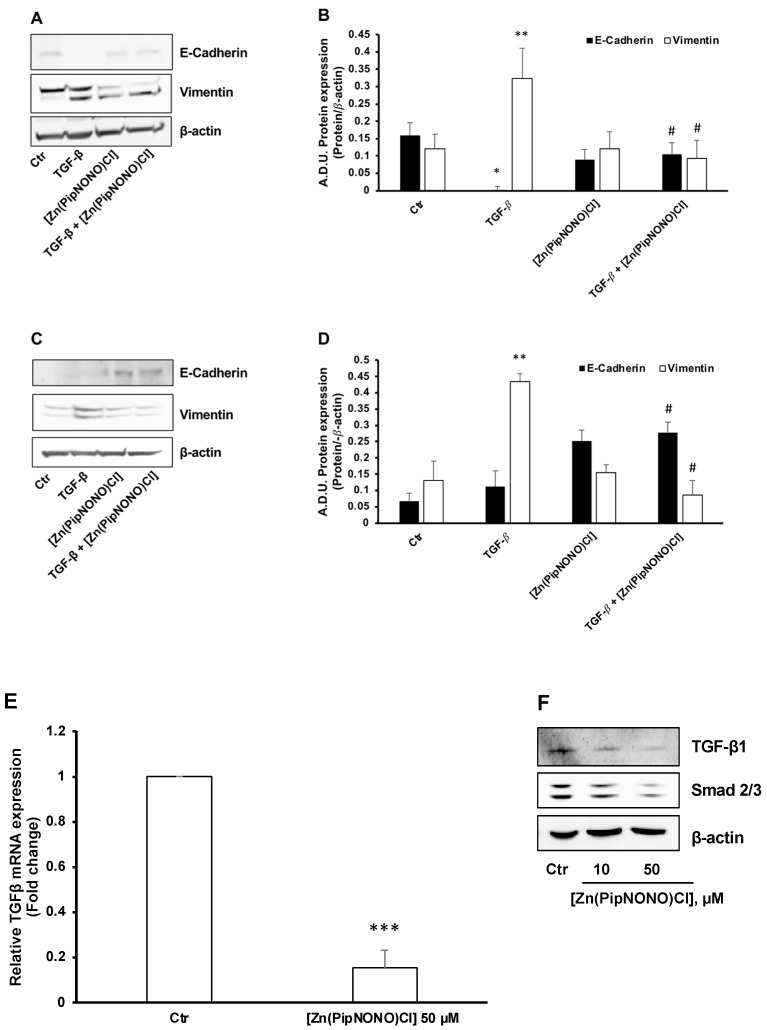
Effect of [Zn(PipNONO)Cl] on EMT induced in tumor cells by exposure to TGF-β1 and on the autocrine TGF-β signaling loop. EMT protein markers were monitored in A549 (**A**,**B**) and A375 (**C**,**D**) cells exposed for 96 h with 10 ng/mL TGF-β1 in the absence and in the presence of 50 µM [Zn(PipNONO)Cl] in medium with 0.5% FBS. * *p* ˂ 0.05 and ** *p* < 0.01 TGF-β1 vs. control (Ctr); # *p* ˂ 0.05 [Zn(PipNONO)Cl] + TGF-β1 vs. TGF-β1 alone. A representative blot of 3 independent experiments is reported. β-actin was used as a loading control. (**E**). TGF-β1 gene expression in A549 treated with [Zn(PipNONO)Cl] for 18 h in medium with 2% serum. Data are reported as fold changes relative to A549 untreated (Ctr), assigned to 1. *** *p* < 0.001 vs. A549 Ctr. (**F**). TGF-β1 and Smad2/3 protein expression in A549 treated with [Zn(PipNONO)Cl] for 18 h in medium with 2% FBS. Representative blots of 3 independent experiments are reported. β-actin was used as a loading control. Whole uncropped blots and densitometry intensity ratio related to panel F are reported in Supplemental material (Figure S1 and Table S1).

TGF-β1 was used as an inductive stimulus of EMT, as reported in the literature [25,53]. Tumor cells exposed to 10 ng/mL TGF-β1 for 96 h underwent the phenomenon of EMT, characterized by elongated fibroblast-like cell phenotype [50]. From a molecular point of view, this led to mesenchymal markers typical of cancer cells being activated, such as vimentin, leading toward an invasive phenotype, while epithelial markers, such as E-cadherin, disappeared (Figure 3A,B for A549 and Figure 3C,D for A375).

In the presence of 50 μM [Zn(PipNONO)Cl], TGF-β1 failed to promote a mesenchymal phenotype. Indeed, tumor cells manifested higher expression of the cell–cell contact protein E-cadherin and reduced vimentin expression (Figure 3A,B for A549 and Figure 3C,D for A375).

As described, autocrine TGF-β1 can feed a positive loop to facilitate EMT in A549 cells [54]. On that basis, we investigated whether [Zn(PipNONO)Cl] could interfere with autocrine TGF-β signaling in A549 cells maintained in medium with 2% FBS. As reported in Figure 3E, the basal transcription of *TGF-β1* was drastically reduced after 18 h of treatment with the Zn-based nonoate (50 μM). Consistently, we found a decrease in TGF-β1 protein expression in a concentration-dependent manner (Figure 3F). Concomitantly, a reduction of Smad2/3, the downstream transcription factor of TGF-β1, was detected in A549 cell treated with the NO donor (Figure 3F).

In conclusion, treatment with [Zn(PipNONO)Cl] significantly reverted the EMT induced by TGF-β1 stimulation of tumor cells, and reduced the endogenous autocrine EMT signaling loop in A549 cells.

### 3.4. Effect of [Zn(PipNONO)Cl] on EndMT in Endothelial Cells Co-Cultured with Tumor Cells

Using a co-culture system set up with the aid of Transwell supports, the EndMT phenomenon was examined, allowing the interaction of tumor cells with HUVECs. In this way, it was possible to evaluate, in endothelial cells, the contributions of tumor cells to the acquisition of a mesenchymal phenotype, and the effect of the treatment with [Zn(PipNONO)Cl].

**Figure 4:**
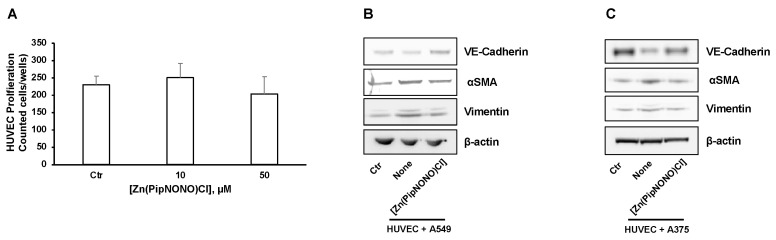

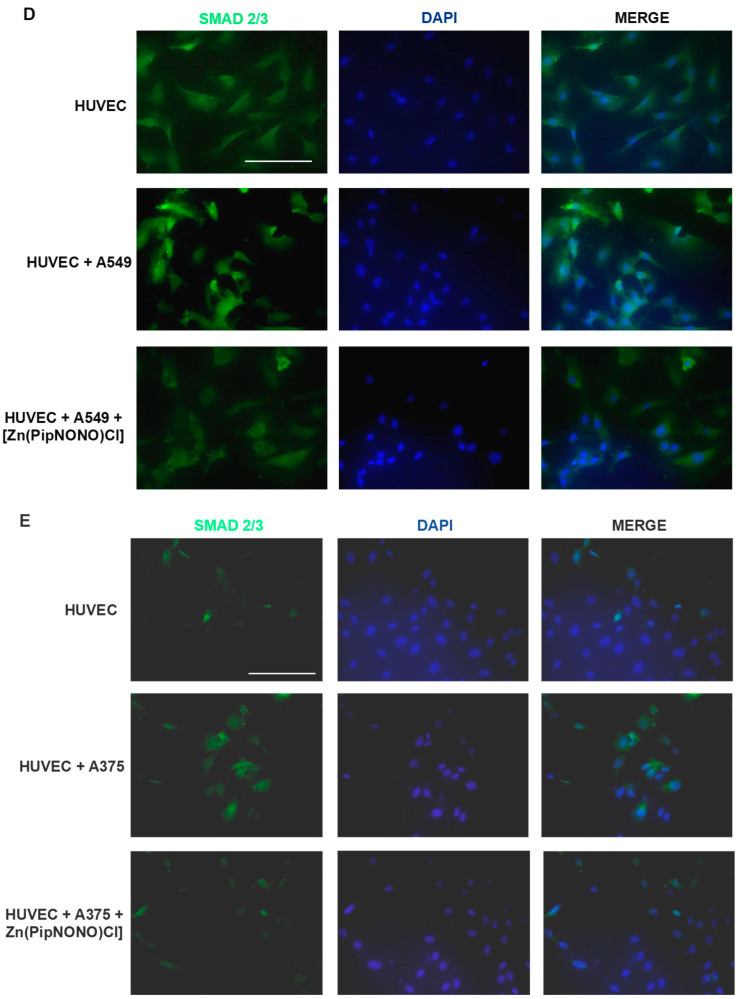
Effects of [Zn(PipNONO)Cl] on EndMT and TGF-β signaling induced by tumor cells co-culture. (**A**) Assessment of the endothelial cell survival following treatment with [Zn(PipNONO)Cl]. This preliminary experiment excluded any toxic effect by 10 and 50 μM concentrations of [Zn(PipNONO)Cl] on HUVECs. (**B**,**C**) Expression of typical markers of endothelial and mesenchymal phenotype in endothelial cells in co-culture with A549 (**B**) and A375 (**C**). The order of loading of the samples was: HUVECs alone (Control, Ctr); HUVECs + tumor cells (cell co-culture); HUVECs + tumor cells + [Zn(PipNONO)Cl] (cell co-culture treated with 10 μM of the metal nonoate). A representative image out of 3 with overlapping results is reported. Whole blots and densitometry readings of the bands are reported in Supplemental material Figure S2 and Table S2. (**D**,**E**) Images of immunostaining for Smad2/3 (green) and DAPI (blue) in HUVECs treated with 2% FBS, co-cultured with A549 (**D**) or A375 (**E**) for 4 days in the absence and in the presence of 10 μM [Zn(PipNONO)Cl]. Images were obtained by using fluorescence microscope (Nikon Eclipse E400 at 40× magnification). Scale bar, 50 μm.

Initially, in order to evaluate the possible cytoxicity in endothelial cells by [Zn(PipNONO)Cl], a cell survival experiment was performed on HUVECs exposed to increasing concentrations of the zinc-based nonoate. As shown in Figure 4A, the concentrations used in the co-culture system (10 and 50 μM) did not change the number of endothelial cells, which is known to be highly sensitive to circulating xenobiotics.

The EndMT phenomenon, characterized by loss of endothelial properties and the acquisition of a mesenchymal phenotype, was studied by Western blot in endothelial cell monolayers exposed to cancer cells in the co-culture system. Our aim was to evaluate whether [Zn(PipNONO)Cl] could mitigate the effect of tumor-derived products on the induction of EndMT. The expression of vimentin and αSMA (mesenchymal markers) and VE-cadherin (typical endothelial marker) was studied on HUVEC lysates. Four days of co-culturing with tumor cells induced an increase in the expression of both vimentin and αSMA in HUVECs. Following the co-culture with [Zn(PipNONO)Cl], these markers were reduced (Figure 4B for A549 and Figure 4C for A375 co-culture). Contrary to this, the expression of VE-cadherin (endothelial marker) reduced in endothelial cells co-cultured with tumor cells, and the treatment of the co-culture with [Zn(PipNONO)Cl] caused an increase in the expression of endothelial marker, restoring the endothelial phenotype to a normal condition (Figure 4B,C).

We therefore examined the effect of the Zn-based nonoate on TGF-β1 signaling, a central player in driving EndMT progression [55]. We evaluated the effect of tumor cells, alone or in combination with [Zn(PipNONO)Cl], on the downstream transcription factors of TGF-β1 and Smad2/3. In particular, to define the cellular localization of Smad2/3, HUVECs were analyzed through immunofluorescence staining. Tumor cells induced positive staining of Smad2/3 in HUVECs, especially localized in the cell nuclei (Figure 4D and Figure 4E for HUVECs co-cultured with A549 and A375, respectively), confirming the above observation on the expression of the upstream tumor-derived TGF-β1 (Figure 3F). In the co-cultures, [Zn(PipNONO)Cl] treatment induced a significant reduction in Smad2/3 expression in endothelial cells, including its nuclear translocation, which was particularly impaired in the A549-containing transwell (Figure 4D and Figure 4E for HUVECs co-cultured with A549 and A375, respectively).

In summary, our results document the protective effects of the zinc-based nonoate on the maintenance of a normal endothelial phenotype, through impairment of TGF-β1 signaling in both tumor and endothelial cells.

## 4. Discussion

Our study reported the inhibitory effect of NO released by the Zn-based metal nonoate [Zn(PipNONO)Cl] on two human tumor models representing NSLC and melanoma. In tumors, the effect of NO varies depending on its specific timing, location and concentration within the tissue [3,4,5,6]. Additionally, the tumor microenvironment and the genetic background of the specific cancer are also important in determining the role of NO as suppressor or promoter [5,56]. The cytotoxic role of NO has been implicated in several studies in which high levels of inducible nitric oxide synthase (iNOS) are associated with tumor cytotoxicity, whereas low levels may promote tumor growth and neovascularization and metastasis [56,57].

Here, we demonstrate the antitumor properties of novel metal nonoate, in particular [Zn(PipNONO)Cl], through the inhibition of EMT and EndMT in melanoma and lung cancer cells used as models. We documented the cytotoxic activity of [Zn(PipNONO)Cl] at doses higher than 100 μM: in the range 10–100 μM, NO inhibited tumor migration and invasiveness, hallmarks of the EMT process.

TGF-β1 is a strong inducer of EMT through loss of epithelial markers and gain of mesenchymal markers [24,25,53]. Tumor cells exposed for 96 h to exogenous TGF-β1 acquired elongated shape. The co-treatment with [Zn(PipNONO)Cl] inhibited the expression of TGF-β1-stimulated EMT markers, maintained the expression of epithelial markers (E-cadherin) and downregulated the mesenchymal one (vimentin). Further, the zinc-based nonoate reduced the autocrine loop of this cytokine, which is critical in cancer malignancy, starting from a transcriptional level.

Our findings suggest that NO can serve as a regulator of EMT by interfering negatively with the expression and activity of TGF-β1. The anti-invasiveness and anti-metastatic properties of NO through the inhibition of angiogenic factors such as FGF-2 and TGFβ-1 have been previously reported [58]. Accordingly, we observed that exposure of tumor cells to [Zn(PipNONO)Cl] counteracted the expression of TGF-β1 and its downstream transcription factor Smad2/3, restoring the epithelial phenotype of tumor cells.

Tumor progression depends also by favorable tumor microenvironment, including endothelial and stromal cells. Here we demonstrated that [Zn(PipNONO)Cl] inhibits the mesenchymal transformation of endothelial cells (EndMT) induced by tumor cells, restoring markers of endothelial integrity (VE-cadherin) and impairing the mesenchymal markers αSMA and vimentin. To the best of our knowledge, the contribution of NO in modulating EndMT is poorly demonstrated in the literature. We previously demonstrated the reacquisition of VE-cadherin and permeability barrier function by [Ni(PipNONO)Cl] in HUVECs exposed to IL-1β [16,59]. Smeda and coworkers demonstrated that NO deficiency in pulmonary endothelium facilitated breast cancer metastasis [60]. In tumor and endothelial cell co-cultures, we have found that HUVECs underwent EndMT, acquiring characteristics of diverse cells, such as fibroblast and smooth muscle cells. The EndMT process is reported to evolve through different subsequent steps [55]. Importantly, we demonstrate that after 96 h of treatment, [Zn(PipNONO)Cl] not only reverted the effect of tumor cells on the declined expression of endothelial markers and on the upregulation of mesenchymal ones, but also neutralized TGF-β1 signaling. Although the molecular mechanisms of NO donors are not well documented, it is plausible that the interference with stemness phenotype could mediate the transition toward mesenchymal ones [61].

We are aware that part of the literature reports on the protumorigenic effect of NO (see [3,4,5,6] for review on contrasting findings on NO in tumor biology). However, how can we conciliate all these findings? Apart the cytotoxic activity of high levels of NO (>200–500 m μM), as seen in this paper—probably due to peroxynitrate production upon interaction with reactive oxygens species—the effect of NO depends on many factors affecting both the NO availability at tissue and cell levels (kinetics of NO release by NO-based drugs; drug availability within the tumor microenvironment; presence of reactive oxygen species and SH moieties or other scavengers during drug distribution) and the target responsiveness (presence of reactive oxygen species and SH moieties or other scavengers within the tumor mass; efficiency of damage repair systems; availability of target protein/enzyme). Other factors that probably influence tumor responsiveness are the presence and quality of the inflammatory environment and the intrinsic ability to produce NO, represented mainly by iNOS whose levels can vary in time and within the tumor mass. We can hypothesize that when the levels of iNOS are high (as in squamous tumors, and colon and prostate cancer) [62,63], the best approach is to use NOS inhibitors, whereas NO-based derivatives can be proposed for tumors with low/moderate levels of iNOS, thereby promoting a reduction in NO levels and inflammation. Indeed, the cellular models used in the present study showed modest expression of iNOS. Based on these concepts, we feel it is appropriate to propose the use of NO donors to control neoplastic progression/aggressiveness for tumors with low–moderate levels of iNOS expression.

Similar considerations can be made for the role of NO in inflammation. While we demonstrated an anti-inflammatory effect (see the inhibition of TGF-β1 signaling and the physiological protective effect of NO in the cardiovascular system), NO has been demonstrated to play a crucial role in the development of chronic inflammation. As example, the presence of NO in cancer may regulate tumor-associated macrophage metabolism and function, favoring the persistence of inflammation in tumorigenesis and cancer stemness [63,64,65,66,67]. These findings appear tissue-specific, since in most of the reports the association high NO levels with inflammation, oxidative status, DNA damage and tumorigenesis has been described for cancer of the gastrointestinal apparatus, upper airways, the reproductive organs and the breast. Again, the time and concentration dependency of NO’s effects (affected on its turn by the expressed NOS isoform) on tumor cells and target tissue propensity to respond to NO and its reactive species can be speculated as responsible for the opposite results [5,66].

Overall, the evaluation of the expression level of NOS isoforms in cancer cells and in specific inflammatory/immune cell subtypes present in the tumor microenvironment in relation to tumor aggressiveness and EMT/EndMT needs to be further assessed both for diagnostic use and personalized treatment choice and to adequately design the immunotherapy/target therapy/chemotherapy combination.

For cancer therapy, the utility of authentic aqueous solutions of NO and most currently available NO donors (represented by low-molecular-weight molecules) is highly limited due to their short half-lives, instability under physiological conditions, fast systemic clearance, unspecific NO release and NO-independent toxicities. The metal nonoates under development in our laboratory have demonstrated safety in mice and rats. In the range 0.5–10 mg/kg/day given i.p., [Ni(PipNONO)Cl] did not affect the blood pressure in normal mice and rats, though reducing hypertension and inflammatory features in SHR after 1 month of repeated administration [16]. Interestingly, even if the kinetics of NO release is in the order of minutes, the long-term protective effect of metal nonoates was presumably due to activation of the eNOS at the vascular level, thereby igniting the endogenous eNOS/NO upregulation and prolonging its kinetics [16,17]. Based on these data, in vivo antitumor efficacy experiments with metal nonoates seem to be feasible.

Since NO acts within the tumor differently over a range of concentrations, and therefore, it must be delivered at certain concentration to tumor cells, the development of stable and tuned NO releasing compounds is mandatory [68]. With the development of nanotechnology, the application of smart biomaterials for the controlled delivery of NO is emerging as one of the promising strategies for tumor targeting [69]. Another approach is represented by the combination of an NO donor with other drugs. There has been an extensive effort to develop hybrid NO donor drugs by attaching NO donor moieties to currently available anticancer drugs. The aim is to maintain the pharmacological activity of the parent drug while gaining benefits from the biological actions of NO [70,71]. Recently, conjugates of NO donors with other targeting drugs, such as antibodies, have been developed to ensure specific targeting to tumor cells and to prevent systemic toxicities. An investigation has been reported that took into consideration the cluster of differentiation 24 (CD24) which is overexpressed in many solid tumors, such as hepatocellular carcinoma, and rarely expressed in normal cells [72].

A further opportunity is the sensitization effect of NO donors on cancer resistant cells to both chemotherapy and immunotherapy. NO can sensitize tumor cells to other therapies by inducing apoptosis, along with the inhibition of cell proliferation, invasion and metastasis, through removal of the immune resistance of cancer cells. NO donors have been shown to have synergistic effects in combination with cancer therapies, such as photodynamic therapy, chemotherapy, radiotherapy and immunotherapy [11]. On top of this, the inhibition of the EMT/EndMT phenotype switch, or its reversion, can provide added value against this multifaced enemy.

## 5. Conclusions

In conclusion, from the in vitro data obtained on cellular models of NSCL and melanoma, the NO donor [Zn(PipNONO)Cl] acts on both tumor and endothelial components, controlling epithelial– and endothelial–mesenchymal transitions and the crosstalk of tumor-endothelial cells within the tumor microenvironment. Exogenously-given NO thus appears a promising therapeutic tool to control tumor growth and metastasis, by acting both on tumor and endothelial cells, reprogramming the cells to return toward typical epithelial and endothelial phenotypes. The recovery and maintenance of endothelial integrity and barrier function are crucial features of normally functioning vessels. Indeed, the endothelial vessel normalization is now seen as an interesting objective in the combination regimens now used for cancer therapy, such as immunotherapies and antiangiogenic strategies, and other combinations [73]. In the near future, NO donors and NO hybrids/conjugates should be continued to be assessed in preclinical and clinical studies based on their promising multitarget potential.

## Data Availability

Data supporting the reported results can be obtained from the corresponding author.

## Supplementary Materials

The following supporting information can be downloaded at: https://www.mdpi.com/article/10.3390/cancers14174240/s1, Figure S1: Whole blots of Figure 3 Western blots; Figure S2: Whole blots of Figure 4 western blots; Table S1: Densitometry readings of Figure 3F blots; Table S2: Densitometry readings of Figure 4B,C blots.

## References

[1] Albina J.E., Reichner J.S. (1998). Role of nitric oxide in mediation of macrophage cytotoxicity and apoptosis. Cancer Metastasis Rev..

[2] Chang C.F., Diers A.R., Hogg N. (2015). Cancer cell metabolism and the modulating effects of nitric oxide. Free Radic. Biol. Med..

[3] Chung H.T., Pae H.O., Choi B.M., Billiar T.R., Kim Y.M. (2001). Nitric oxide as a bioregulator of apoptosis. Biochem. Biophys. Res. Commun..

[4] Choudhari S.K., Chaudhary M., Bagde S., Gadbail A.R., Joshi V. (2013). Nitric oxide and cancer: A review. World J. Surg. Oncol..

[5] Khan F.H., Dervan E., Bhattacharyya D.D., McAuliffe J.D., Miranda K.M., Glynn S.A. (2020). The Role of Nitric Oxide in Cancer: Master Regulator or NOt?. Int. J. Mol. Sci..

[6] Huerta S., Chilka S., Bonavida B. (2008). Nitric oxide donors: Novel cancer therapeutics (review). Int. J. Oncol..

[7] Alimoradi H., Greish K., Gamble A.B., Giles G.I. (2019). Controlled Delivery of Nitric Oxide for Cancer Therapy. Pharm. Nanotechnol..

[8] Xu L., Xie K., Fidler I.J. (1998). Therapy of human ovarian cancer by transfection with the murine interferon beta gene: Role of macrophage-inducible nitric oxide synthase. Hum. Gene Ther..

[9] Qiu M., Chen L., Tan G., Ke L., Zhang S., Chen H., Liu J. (2015). A reactive oxygen species activation mechanism contributes to JS-K-induced apoptosis in human bladder cancer cells. Sci. Rep..

[10] Bonavida B., Baritaki S., Huerta-Yepez S., Vega M.I., Chatterjee D., Yeung K. (2008). Novel therapeutic applications of nitric oxide donors in cancer: Roles in chemo- and immunosensitization to apoptosis and inhibition of metastases. Nitric Oxide.

[11] Bonavida B. (2020). Sensitizing activities of nitric oxide donors for cancer resistance to anticancer therapeutic drugs. Biochem. Pharmacol..

[12] Hirst D., Robson T. (2007). Targeting nitric oxide for cancer therapy. J. Pharm. Pharmacol..

[13] Riganti C., Miraglia E., Viarisio D., Costamagna C., Pescarmona G., Ghigo D., Bosia A. (2005). Nitric oxide reverts the resistance to doxorubicin in human colon cancer cells by inhibiting the drug efflux. Cancer Res..

[14] Li B., Ming Y., Liu Y., Xing H., Fu R., Li Z., Ni R., Li L., Duan D., Xu J. (2020). Recent Developments in Pharmacological Effect, Mechanism and Application Prospect of Diazeniumdiolates. Front. Pharmacol..

[15] Ziche M., Donnini S., Morbidelli L., Monzani E., Roncone R., Gabbini R., Casella L. (2008). Nitric oxide releasing metal-diazeniumdiolate complexes strongly induce vasorelaxation and endothelial cell proliferation. ChemMedChem.

[16] Monti M., Ciccone V., Pacini A., Roggeri R., Monzani E., Casella L., Morbidelli L. (2016). Anti-hypertensive property of a nickel-piperazine/NO donor in spontaneously hypertensive rats. Pharmacol. Res..

[17] Monti M., Hyseni I., Pacini A., Monzani E., Casella L., Morbidelli L. (2018). Cross-talk between endogenous H2S and NO accounts for vascular protective activity of the metal-nonoate Zn(PipNONO)Cl. Biochem. Pharmacol..

[18] Ciccone V., Monti M., Monzani E., Casella L., Morbidelli L. (2018). The metal-nonoate Ni(SalPipNONO) inhibits in vitro tumor growth, invasiveness and angiogenesis. Oncotarget.

[19] Dongre A., Weinberg R.A. (2019). New insights into the mechanisms of epithelial–mesenchymal transition and implications for cancer. Nat. Rev. Mol. Cell. Biol..

[20] Platel V., Faure S., Corre I., Clere N. (2019). Endothelial-to-Mesenchymal Transition (EndoMT): Roles in Tumorigenesis, Metastatic Extravasation and Therapy Resistance. J. Oncol..

[21] Kalluri R., Weinberg R.A. (2009). The basics of epithelial mesenchymal transition. J. Clin. Investig..

[22] Tam W.L., Weinberg R.A. (2013). The epigenetics of epithelial-mesenchymal plasticity in cancer. Nat. Med..

[23] Yang J., Antin P., Berx G., Blanpain C., Brabletz T., Bronner M., Campbell K., Cano A., Casanova J., Christofori G. (2020). Guidelines and definitions for research on epithelial-mesenchymal transition. Nat. Rev. Mol. Cell Biol..

[24] Massague J. (2012). TGFβ signalling in context. Nat. Rev. Mol. Cell Biol..

[25] Stuelten C.H., Zhang Y.E. (2021). Transforming Growth Factor-β: An Agent of Change in the Tumor Microenvironment. Front. Cell Dev. Biol..

[26] Bonavida B., Baritaki S. (2011). Dual role of NO donors in the reversal of tumor cell resistance and EMT: Downregulation of the NF-κB/Snail/YY1/RKIP circuitry. Nitric Oxide.

[27] Pan X., Wang X., Lei W., Min L., Yang Y., Wang X., Song J. (2009). Nitric oxide suppresses transforming growth factor-β1-induced epithelial-to mesenchymal transition and apoptosis in mouse hepatocytes. Hepatology.

[28] Powan P., Chanvorachote P. (2014). Nitric oxide mediates cell aggregation and mesenchymal to epithelial transition in anoikis-resistant lung cancer cells. Mol. Cell. Biochem..

[29] Maleszewska M., Moonen J.R., Huijkman N., van de Sluis B., Krenning G., Harmsen M.C. (2013). IL-1beta and TGFbeta2 synergistically induce endothelial to mesenchymal transition in an NFkappaB-dependent manner. Immunobiology.

[30] Bischoff J. (2019). Endothelial-to-Mesenchymal Transition Purposeful Versus Maladaptive Differentiation. Circ. Res..

[31] Huang Q., Gan Y., Yu Z., Wu H., Zhong Z. (2021). Endothelial to Mesenchymal Transition: An Insight in Atherosclerosis. Front. Cardiovasc. Med..

[32] Chen P.Y., Qin L., Barnes C., Charisse K., Yi T., Zhang X., Ali R., Medina P.P., Yu J., Slack F.J. (2012). FGF regulates TGF-beta signaling and endothelial-to-mesenchymal transition via control of let-7 miRNA expression. Cell Rep..

[33] Krenning G., Moonen J.R., van Luyn M.J., Harmsen M.C. (2008). Vascular smooth muscle cells for use in vascular tissue engineering obtained by endothelial-to-mesenchymal transdifferentiation (EnMT) on collagen matrices. Biomaterials.

[34] Li Y., Lui K.O., Zhou B. (2018). Reassessing endothelial-to-mesenchymal transition in cardiovascular diseases. Nat. Rev. Cardiol..

[35] Zeisberg E.M., Potenta S., Xie L., Zeisberg M., Kalluri R. (2007). Discovery of endothelial to mesenchymal transition as a source for carcinoma-associated fibroblasts. Cancer Res..

[36] Gasparics Á., Rosivall L., Krizbai I.A., Sebe A. (2016). When the endothelium scores an own goal: Endothelial cells actively augment metastatic extravasation through endothelial-mesenchymal transition. Am. J. Physiol. Heart Circ. Physiol..

[37] Anderberg C., Cunha S.I., Zhai Z., Cortez E., Pardali E., Johnson J.R., Franco M., Páez-Ribes M., Cordiner R., Fuxe J. (2013). Deficiency for endoglin in tumor vasculature weakens the endothelial barrier to metastatic dissemination. J. Exp. Med..

[38] Xiao L., Dudley A.C. (2017). Fine-tuning vascular fate during endothelial-mesenchymal transition. J. Pathol..

[39] Hutchinson B.D., Shroff G.S., Truong M.T., Ko J.P. (2019). Spectrum of Lung Adenocarcinoma. Semin. Ultrasound CT MRI.

[40] Rambow F., Marine J.C., Goding C.R. (2019). Melanoma plasticity and phenotypic diversity: Therapeutic barriers and opportunities. Genes Dev..

[41] Zafeiriadou A., Kollias I., Londra T., Tsaroucha E., Georgoulias V., Kotsakis A., Lianidou E., Markou A. (2022). Metabolism-Related Gene Expression in Circulating Tumor Cells from Patients with Early Stage Non-Small Cell Lung Cancer. Cancers.

[42] Murtas D., Maxia C., Diana A., Pilloni L., Corda C., Minerba L., Tomei S., Piras F., Ferreli C., Perra M.T. (2017). Role of epithelial-mesenchymal transition involved molecules in the progression of cutaneous melanoma. Histochem. Cell Biol..

[43] Kim S.-H., Song Y., Seo H.R. (2019). GSK-3β regulates the endothelial-to-mesenchymal transition via reciprocal crosstalk between NSCLC cells and HUVECs in multicellular tumor spheroid models. J. Exp. Clin. Cancer Res..

[44] Yeon J.H., Jeong H.E., Seo H., Cho S., Kim K., Na D., Chung S., Park J., Choi N., Kang J.Y. (2018). Cancer-derived exosomes trigger endothelial to mesenchymal transition followed by the induction of cancer-associated fibroblasts. Acta Biomater..

[45] Munaweera I., Shi Y., Koneru B., Patel A., Dang M.H., Di Pasqua A.J., Balkus K.J. (2015). Nitric oxide- and cisplatin-releasing silica nanoparticles for use against non-small cell lung cancer. J. Inorg. Biochem..

[46] Arrieta O., Blake M., de la Mata-Moya M.D., Corona F., Turcott J., Orta D., Alexander-Alatorre J., Gallardo-Rincón D. (2014). Phase II study. Concurrent chemotherapy and radiotherapy with nitroglycerin in locally advanced non-small cell lung cancer. Radiother. Oncol..

[47] Ferraz L.S., Watashi C.M., Colturato-Kido C., Pelegrino M.T., Paredes-Gamero E.J., Weller R.B., Seabra A.B., Rodrigues T. (2018). Antitumor Potential of S-Nitrosothiol-Containing Polymeric Nanoparticles against Melanoma. Mol. Pharm..

[48] Bai C., Xue R., Wu J., Lv T., Luo X., Huang Y., Gong Y., Zhang H., Zhang Y., Huang Z. (2017). O2-(6-Oxocyclohex-1-en-1-yl)methyl diazen-1-ium-1,2-diolates: A new class of nitric oxide donors activatable by GSH/GSTπ with both anti-proliferative and anti-metastatic activities against melanoma. Chem. Commun..

[49] Ciccone V., Zazzetta M., Morbidelli L. (2019). Comparison of the Effect of Two Hyaluronic Acid Preparations on Fibroblast and Endothelial Cell Functions Related to Angiogenesis. Cells.

[50] Ciccone V., Terzuoli E., Ristori E., Filippelli A., Ziche M., Morbidelli L., Donnini S. (2022). ALDH1A1 overexpression in melanoma cells promotes tumor angiogenesis by activating the IL-8/Notch signaling cascade. Int. J. Mol. Med..

[51] Martelli A., Piragine E., Gorica E., Citi V., Testai L., Pagnotta E., Lazzeri L., Pecchioni N., Ciccone V., Montanaro R. (2021). The H2S-Donor Erucin Exhibits Protective Effects against Vascular Inflammation in Human Endothelial and Smooth Muscle Cells. Antioxidants.

[52] Ciccone V., Filippelli A., Angeli A., Supuran C.T., Morbidelli L. (2020). Pharmacological Inhibition of CA-IX Impairs Tumor Cell Proliferation, Migration and Invasiveness. Int. J. Mol. Sci..

[53] Hao Y., Baker D., Ten Dijke P. (2019). TGF-β-Mediated Epithelial-Mesenchymal Transition and Cancer Metastasis. Int. J. Mol. Sci..

[54] Elmansuri A.Z., Tanino M.A., Mahabir R., Wang L., Kimura T., Nishihara H., Kinoshita I., Dosaka-Akita H., Tsuda M., Tanaka S. (2016). Novel signaling collaboration between TGF-β and adaptor protein Crk facilitates EMT in human lung cancer. Oncotarget.

[55] Dejana E., Hirschi K.K., Simons M. (2017). The molecular basis of endothelial cell plasticity. Nat. Commun..

[56] Vannini F., Kashfi K., Nath N. (2015). The dual role of iNOS in cancer. Redox Biol..

[57] Morbidelli L. (2016). Therapeutic Potential of Nitric Oxide Donors in Cancer: Focus on Angiogenesis. Crit. Rev. Oncog..

[58] Baritaki S., Huerta-Yepez S., Sahakyan A., Karagiannides I., Bakirtzi K., Jazirehi A., Bonavida B. (2010). Mechanisms of nitric oxide-mediated inhibition of EMT in cancer: Inhibition of the metastasis-inducer Snail and induction of the metastasis-suppressor RKIP. Cell Cycle.

[59] Monti M., Solito R., Puccetti L., Pasotti L., Roggeri R., Monzani E., Casella L., Morbidelli L. (2014). Protective effects of novel metal-nonoates on the cellular components of the vascular system. J. Pharmacol. Exp. Ther..

[60] Smeda M., Kieronska A., Adamski M.G., Proniewski B., Sternak M., Mohaissen T., Przyborowski K., Derszniak K., Kaczor D., Stojak M. (2018). Nitric oxide deficiency and endothelial-mesenchymal transition of pulmonary endothelium in the progression of 4T1 metastatic breast cancer in mice. Breast Cancer Res..

[61] Waheed S., Cheng R.Y., Casablanca Y., Maxwell G.L., Wink D.A., Syed V. (2019). Nitric Oxide Donor DETA/NO Inhibits the Growth of Endometrial Cancer Cells by Upregulating the Expression of RASSF1 and CDKN1A. Molecules.

[62] Gallo O., Masini E., Morbidelli L., Franchi A., Fini-Storchi I., Vergari W.A., Ziche M. (1998). Role of nitric oxide in angiogenesis and tumor progression in head and neck cancer. J. Natl. Cancer Inst..

[63] Drehmer D., Luiz J.P.M., Hernandez C.A.S., Alves-Filho J.C., Hussell T., Townsend P.A., Moncada S. (2022). Nitric oxide favours tumour-promoting inflammation through mitochondria-dependent and -independent actions on macrophages. Redox Biol..

[64] Puglisi M.A., Cenciarelli C., Tesori V., Cappellari M., Martini M., Di Francesco A.M., Giorda E., Carsetti R., Ricci-Vitiani L., Gasbarrini A. (2015). High nitric oxide production, secondary to inducible nitric oxide synthase expression, is essential for regulation of the tumour-initiating properties of colon cancer stem cells. J. Pathol..

[65] Lin K., Baritaki S., Vivarelli S., Falzone L., Scalisi A., Libra M., Bonavida B. (2022). The Breast Cancer Protooncogenes HER2, BRCA1 and BRCA2 and Their Regulation by the iNOS/NOS2 Axis. Antioxidants.

[66] D’Este F., Della Pietra E., Badillo Pazmay G.V., Xodo L.E., Rapozzi V. (2020). Role of nitric oxide in the response to photooxidative stress in prostate cancer cells. Biochem. Pharmacol..

[67] Pacova H., Astl J., Martinek J. (2009). The pathogenesis of chronic inflammation and malignant transformation in the human upper airways: The role of beta-defensins, eNOS, cell proliferation and apoptosis. Histol. Histopathol..

[68] Mintz J., Vedenko A., Rosete O., Shah K., Goldstein G., Hare J.M., Ramasamy R., Arora H. (2021). Current Advances of Nitric Oxide in Cancer and Anticancer Therapeutics. Vaccines.

[69] Lee J., Hlaing S.P., Hasan N., Kwak D., Kim H., Cao J., Yoon I.S., Yun H., Jung Y., Yoo J.W. (2021). Tumor-Penetrable Nitric Oxide-Releasing Nanoparticles Potentiate Local Antimelanoma Therapy. ACS Appl. Mater. Interfaces.

[70] Dallavalle S., Dobričić V., Lazzarato L., Gazzano E., Machuqueiro M., Pajeva I., Tsakovska I., Zidar N., Fruttero R. (2020). Improvement of conventional anti-cancer drugs as new tools against multidrug resistant tumors. Drug Resist. Updates.

[71] Chegaev K., Riganti C., Lazzarato L., Rolando B., Guglielmo S., Campia I., Fruttero R., Bosia A., Gasco A. (2011). Nitric oxide donor doxorubicins accumulate into Doxorubicin-resistant human colon cancer cells inducing cytotoxicity. ACS Med. Chem. Lett..

[72] Sun F., Wang Y., Luo X., Ma Z., Xu Y., Zhang X., Lv T., Zhang Y., Wang M., Huang Z. (2019). Anti-CD24 Antibody-Nitric Oxide Conjugate Selectively and Potently Suppresses Hepatic Carcinoma. Cancer Res..

[73] Augustin H.G., Koh G.Y. (2022). Antiangiogenesis: Vessel Regression, Vessel Normalization, or Both?. Cancer Res..

