# Supplementary material for: The Nitric Oxide Donor [Zn(PipNONO)Cl] Exhibits Antitumor Activity through Inhibition of Epithelial and Endothelial Mesenchymal Transitions
Source: Cancers (Basel). 2022 Aug 31;14(17):4240. doi: 10.3390/cancers14174240 (PMC9454450; doi:10.3390/cancers14174240)
Supplement: Supplementary file 1 [file cancers-14-04240-s001.zip › cancers-1865511-supplementary.pdf]

Figure S1: Whole blots of Figure 3 Western blots

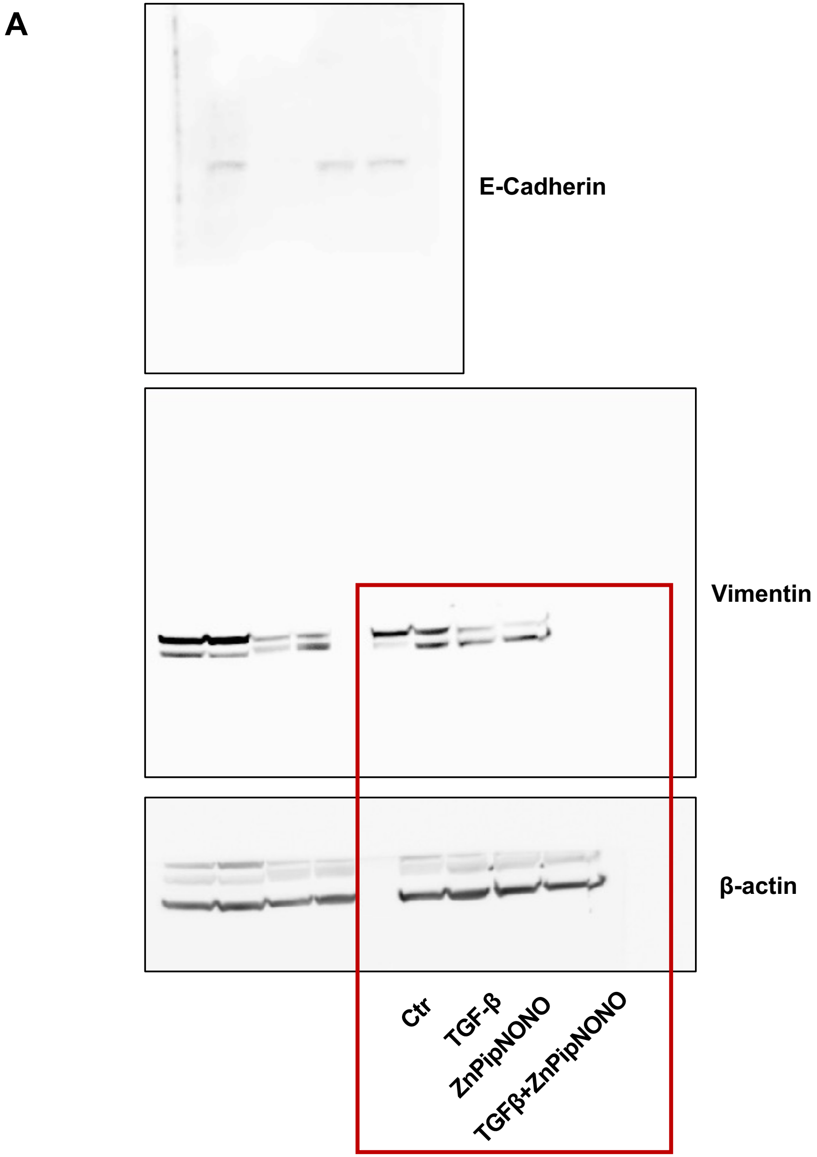

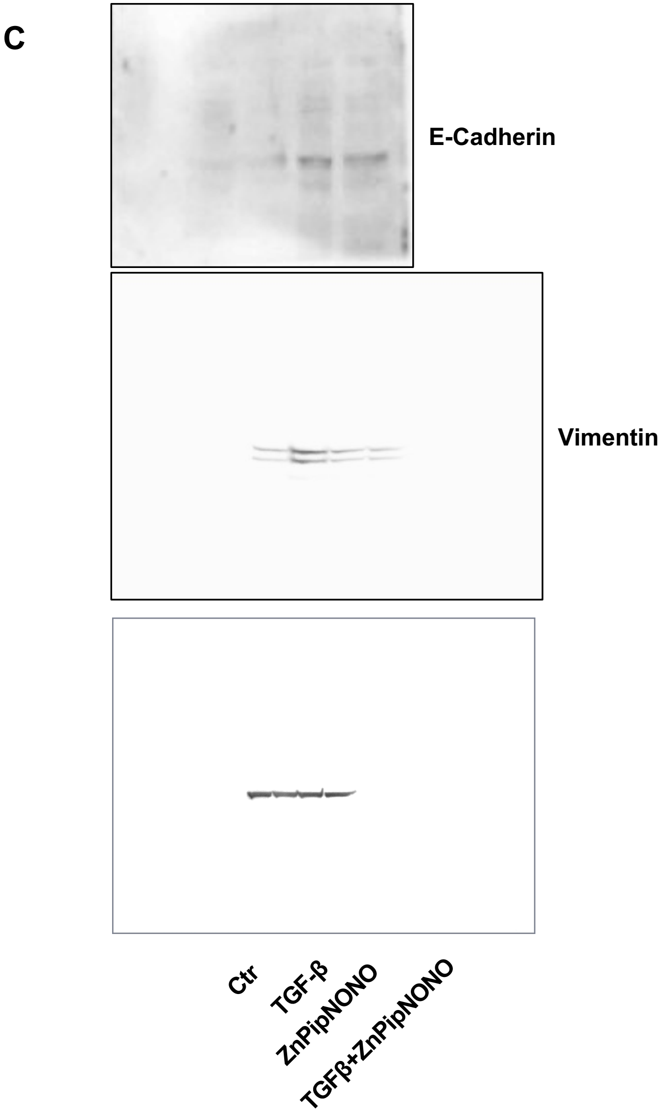

F

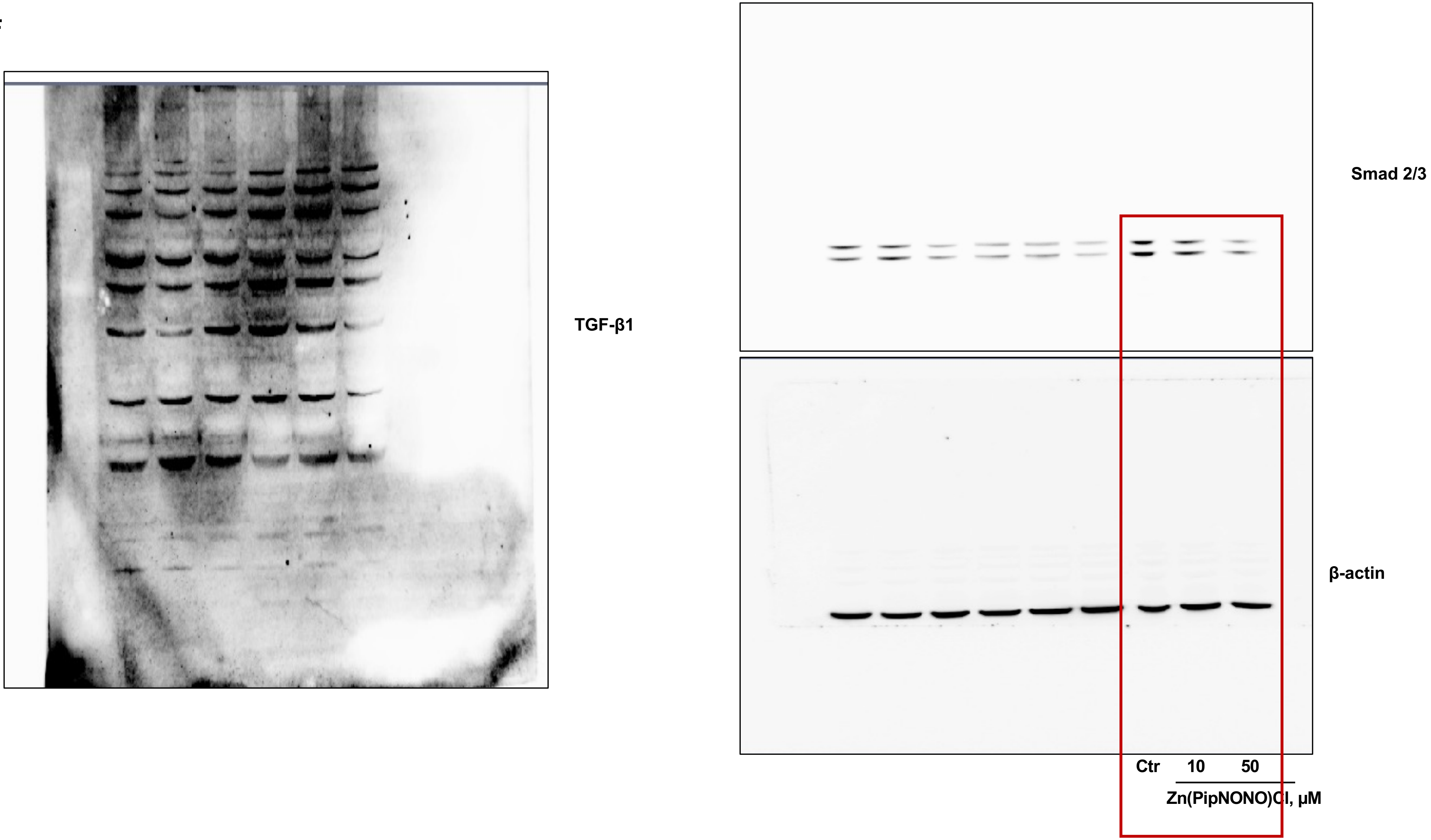

Figure S2
Whole blots of Figure 4 western blots

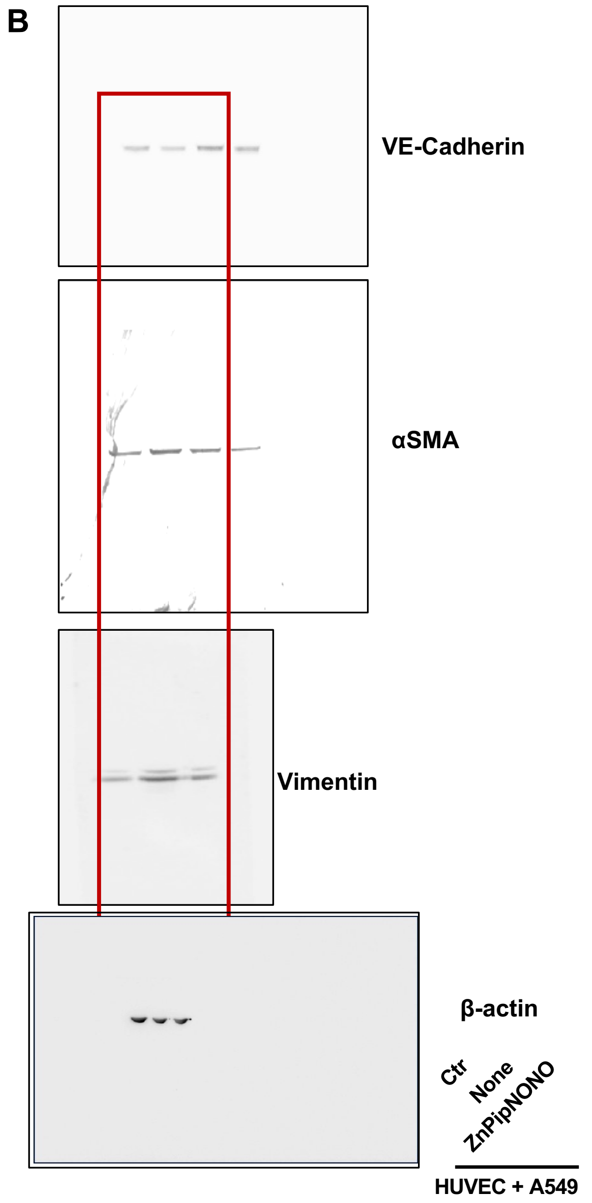

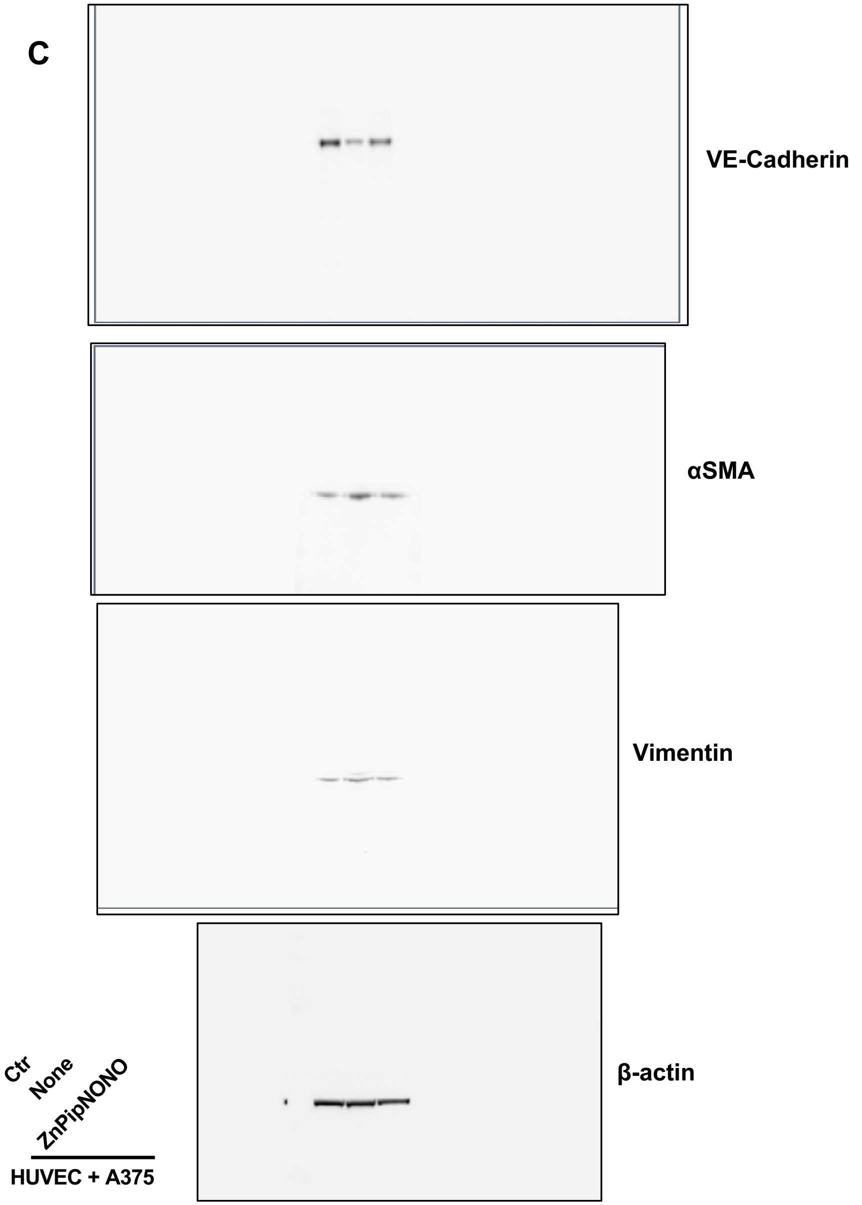

Table S1: Densitometry readings of Figure 3F blots

**Protein of interest**	**Exp. Condition**		
**TGFbeta1**		Mean ADU	SD
	Ctr	0,46778	0,0543
	None	0,0908668	0,00546
	Zn(PipNONO)Cl	0,01308197	0,00476
**Smad2/3**			
	Ctr	0,68325356	0,0898
	None	0,39597666	0,0551
	Zn(PipNONO)Cl	0,20311298	0,0423

Table S2: Densitometry readings of Figure 4 B and C blots

Figure 4B

**VE-cadherin**	Exp. Condition	Mean ADU	SD
	Ctr	0,18105967	0,109
	None	0,10818198	0,0198
	Zn(PipNONO)Cl	0,38266185	0,0498
**Alpha SMA**			
	Ctr	0,36696162	0,04102
	None	0,6035438	0,0872
	Zn(PipNONO)Cl	0,43031341	0,0771
**Vimentin**			
	Ctr	0,21166947	0,031
	None	0,63425393	0,0892
	Zn(PipNONO)Cl	0,33007394	0,0433

Figure 4 C

**VE-cadherin**	Exp. Condition	Mean ADU	SD
	Ctr	0,66524836	0,078
	None	0,21631461	0,0394
	Zn(PipNONO)Cl	0,54317894	0,064
**Alpha SMA**			
	Ctr	0,16036615	0,0283
	None	0,34417272	0,042
	Zn(PipNONO)Cl	0,23547147	0,031
**Vimentin**			
	Ctr	0,11951623	0,0201
	None	0,2061334	0,01983
	Zn(PipNONO)Cl	0,16064768	0,0293
